# Predictors of neurological recovery in spinal epidural abscess: the impact of radiographic compression, ASIA scores, and surgical delay

**DOI:** 10.3389/fcimb.2026.1831170

**Published:** 2026-08-12

**Authors:** Wentao Zhao, Yongrui Yang, Wenkai Ruan, Jianlong Li, Rongpan Dang, Huigang An, Liang Xu, Lu Xing, Yingxin Zhao, Hongdong Tan, Chenggui Zhang

**Affiliations:** Department of Spinal Infection Surgery, Public Health Clinical Center Affiliated to Shandong University, Jinan, Shandong, China

**Keywords:** Asia, magnetic resonance imaging, neurological outcome, spinal epidural abscess, surgical timing

## Abstract

**Background:**

Controversy remains whether spinal epidural abscess (SEA) should be managed conservatively or by early surgical decompression. It is essential to understand how clinical and radiological parameters correlate with neurological deficits and how they predict recovery trajectories.

**Objective:**

Our aim was to evaluate whether the preoperative magnetic resonance imaging (MRI) features, the clinical management factors, and the neurological status on admission can predict neurological recovery in patients with SEA.

**Methods:**

We retrospectively analyzed patients diagnosed with SEA between 2021 and 2024 with a minimum follow-up of 12 months. Collected variables included demographics, duration of preoperative antibiotic therapy, the American Spinal Injury Association (ASIA) motor scores, and the Numerical Rating Scale (NRS) pain scores. Quantitative MRI parameters were assessed, including cord T2 hyperintensity, ossification of the ligamentum flavum (OLF), and maximum spinal cord compression (MSCC). Neurological recovery was calculated using the ASIA recovery rate, which was dichotomized as good (≥50%) or poor (<50%). Logistic regression and receiver operating characteristic (ROC) analyses were performed.

**Results:**

A total of 125 patients were included, of whom 24 were treated conservatively and 101 underwent surgical decompression. Cord T2 hyperintensity (*β* = −13.79, *p* < 0.01), OLF (*β* = −16.59, *p* < 0.01), >50% contrast enhancement (*β* = −8.64, *p* = 0.002), and MSCC (*β* = −0.3, *p* < 0.001) were significantly correlated with worse admission ASIA scores. Cord T2 hyperintensity (*β* = 0.37, *p* = 0.038) and MSCC (*β* = −0.01, *p* = 0.043) were also associated with higher admission NRS scores. Prolonged preoperative antibiotic therapy exceeding 3 weeks was associated with poorer neurological recovery (*p* = 0.04). An admission ASIA score of >88 demonstrated excellent prognostic performance for good recovery (AUC = 0.927, 95%CI = 0.876–0.977; sensitivity = 0.81, specificity = 0.925).

**Conclusion:**

Both the clinical and radiological parameters upon presentation are predictors of neurological recovery rates in SEA. Delayed surgical decompression beyond 3 weeks is associated with poorer neurological recovery.

**Clinical trial registration:**

[website], identifier GWLCZXEC-SOP-K-2025-137.

## Introduction

First described by Morgagni in 1796, spinal epidural abscess (SEA) is an uncommon but potentially catastrophic condition characterized by purulent infection within the epidural space. If left untreated, SEA can rapidly progress to irreversible neurological injury, systemic sepsis, and death. Despite advances in imaging and antimicrobial therapy, the incidence of SEA has increased over recent decades, largely driven by aging populations, increased spinal interventions, diabetes, and immunosuppression (Reihsaus et al., 2000; Darouiche, 2006; Sendi et al., 2007; Arko et al., 2014; Vakili and Crum-Cianflone, 2017).

In light of this background, the current management strategies for SEA include prolonged intravenous antibiotic therapy and surgical decompression with abscess evacuation. While early surgery is widely recommended for patients presenting with neurological deficits, spinal instability, or sepsis, conservative treatment has been advocated in carefully selected cases (Harrington et al., 2001; Parkinson and Sekhon, 2004; Siddiq et al., 2004; Darouiche, 2010; Felício et al., 2011; Kim et al., 2014; Luo et al., 2018; Behmanesh et al., 2020). Consequently, the optimal timing and indication for surgery remain subjects of ongoing debate.

Despite these evolving treatment paradigms, numerous studies have attempted to identify prognostic factors that influence the neurological outcomes in SEA, including the duration of symptoms, the baseline neurological status, and the extent of infection on magnetic resonance imaging (MRI) (Arvin et al., 2013; Sun et al., 2015; Kato et al., 2018). However, there is a lack of consistent and reproducible radiological thresholds capable of stratifying neurological recovery trajectories. In particular, quantitative MRI-based parameters such as spinal canal compromise and spinal cord compression have not been systematically evaluated in this population (Curry et al., 2005).

## Materials and methods

### Study design and patient selection

This is a single-center, retrospective cohort study conducted at Public Health Clinical Center Affiliated with Shandong University. We reviewed the electronic medical records of all patients diagnosed with SEA between January 2021 and December 2024. The inclusion criteria were: 1) radiologically confirmed SEA on MRI; 2) availability of complete clinical, radiological, and follow-up data; and 3) minimum follow-up duration of 1 year. Patients with prior spinal surgery at the involved level, concurrent spinal tumors, or traumatic spinal injuries were excluded.

Although there is no universally accepted cutoff for the duration of preoperative antibiotic therapy in SEA, prolonged conservative management without surgical decompression has been associated with higher failure rates and an increased risk of neurological deterioration. In clinical practice, a total antibiotic course of 4–8 weeks is commonly recommended. However, several studies have suggested that delayed surgical intervention beyond the early phase may compromise neurological recovery (Ghobrial et al., 2014; Behmanesh et al., 2020; Chae et al., 2021). Surgical timing delay was defined as the time interval from MRI confirmation of SEA to the date of surgical decompression. In the surgical cohort, the duration of preoperative antibiotic therapy was calculated from the initiation of intravenous antibiotics to the day of surgery. A threshold of preoperative antibiotic therapy exceeding 3 weeks (>21 days) was selected to represent delayed surgical intervention.

Based on these considerations, as well as the distribution of treatment timing within our cohort, a duration of preoperative antibiotic therapy exceeding 3 weeks was selected as a clinically meaningful threshold to represent delayed surgical intervention. This cutoff was chosen to balance clinical relevance and sample size considerations and to reflect a period during which ongoing spinal cord compression and inflammatory insult may persist despite antimicrobial therapy.

The study was reviewed by the Institutional Review Board (IRB) of Shandong Public Health Clinical Center, Shandong University. Due to its retrospective nature, with no additional risk to participants and with the use of de-identified data, the IRB waived the requirement for informed consent (IRB waiver no. [GWLCZXEC-SOP-K-2025-137]). The study was conducted in full accordance with the Declaration of Helsinki and local ethical regulations.

### Data collection

The demographic and clinical variables included age, gender, preoperative antibiotic therapy days, and treatment modality (conservative *vs*. surgical). Neurological function was assessed using the American Spinal Injury Association (ASIA) motor score, which evaluates strength in 10 key muscle groups bilaterally on a scale from 0 (no contraction) to 5 (normal strength), yielding a total possible score of 0–100. Assessments were recorded at admission and during follow-up visits. In this retrospective study, examiners were not formally blinded to the imaging findings or treatment details as the evaluations were performed as part of standard clinical care.

This approach is part of the International Standards for Neurological Classification of Spinal Cord Injury, which is widely accepted as an objective measure of neurological impairment in spinal cord injuries (Graves et al., 2006; Rupp et al., 2021), and the Numerical Rating Scale (NRS) for pain, which were recorded both on admission and at each follow-up.

### Radiological assessment

Preoperative MRI data were independently reviewed by two experienced radiologists blinded to the clinical outcomes. The following radiological parameters were quantified: cord T2 hyperintensity, ossification of the ligamentum flavum (OLF), and contrast enhancement extent, categorized into ≤50% or >50% of the abscess volume. Maximum spinal cord compression (MSCC) was calculated according to previously described methods on mid-sagittal MRI (Aarabi et al., 2011). The length of abscess was measured as the longest dimension on sagittal MRI (in millimeters). The MSCC area (in square millimeters) was measured at the level of greatest cord compression on axial MRI. Discrepancies in the radiological interpretations were resolved by consensus. Inter-observer agreement was assessed using Cohen’s kappa coefficient for categorical variables (0.88).

### Outcome measures

The primary outcome was neurological recovery, defined as the change in ASIA motor score from admission to the 12-month follow-up. The ASIA recovery rate was calculated using the following formula: (Final follow-up ASIA score − Admission ASIA score)/(100 − Admission ASIA score) * 100%. ΔASIA = ASIA_final_ − ASIA_admission_. The ASIA recovery rates were further dichotomized into “good” (≥50% improvement from baseline deficit) or “poor” (<50% improvement). Secondary outcomes included pain improvement (i.e., a reduction in the NRS score) at the final follow-up.

### Statistical analysis

SPSS software (version 22.0; SPSS Incorporated, Chicago, IL, USA) was used for data analysis. Continuous variables are expressed as the mean ± standard deviation (SD), whereas categorical variables are presented as frequencies and percentages. Independent *t*-tests were used to compare continuous variables, and chi-square tests were used for categorical variables. Univariate linear regression analyses were performed to assess associations between individual variables and neurological outcomes.

**Figure 1:**
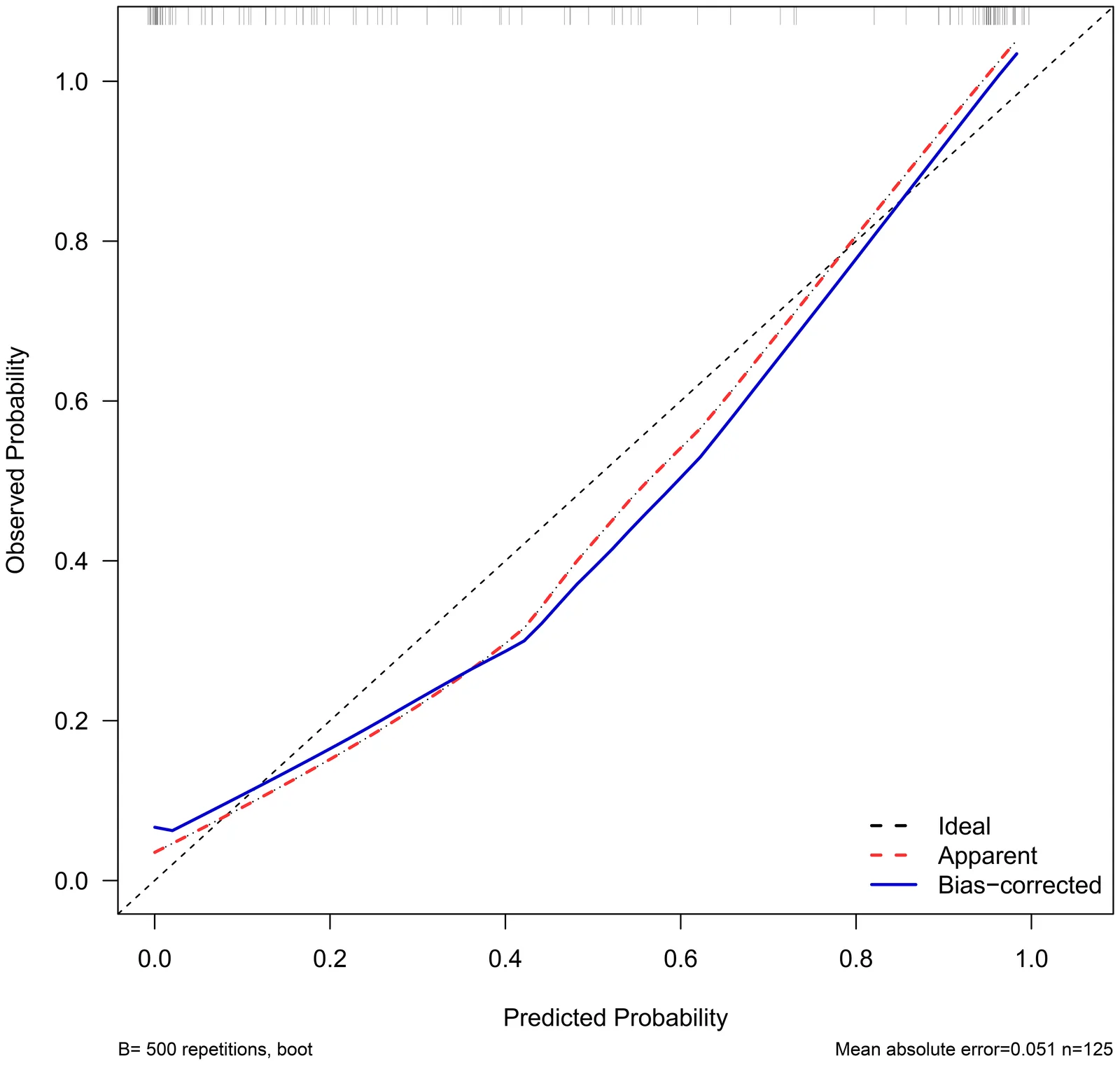
Bootstrap validation of the multivariable logistic regression model for good American Spinal Injury Association (ASIA) recovery.

For the binary outcome of good *versus* poor ASIA recovery (≥50% *vs*. <50%), candidate variables with *p* < 0.05 in the univariate analysis were first entered into LASSO (least absolute shrinkage and selection operator) penalized logistic regression for variable selection. The optimal penalty parameter (*λ*) was determined using the minimum lambda based on 10-fold cross-validation. The variables retained by the LASSO model were then included in the final multivariable binary logistic regression. Model performance and stability were internally validated using 500 bootstrap resamples, with the optimism-corrected area under the receiver operating characteristic curve (AUC) and 95% confidence intervals (CIs) reported (**Figure 1**). AUC was calculated to evaluate the discriminatory ability for good ASIA recovery. Multicollinearity was assessed using variance inflation factors (VIFs). Statistical significance was set at *p* < 0.05. No missing data were present in the final dataset.

## Results

### Patient demographics

A total of 125 patients meeting the inclusion criteria were analyzed. The cohort had a mean age of 63.54 ± 12.2 years, with 73 (58.4%) male and 52 (41.6%) female patients. There were 24 patients (19.2%) treated conservatively with targeted antibiotics, while 101 patients (80.8%) underwent surgical decompression. The baseline demographic and clinical characteristics are summarized in **Table 1**. The average duration from admission to the final follow-up was 22 ± 9.5 months (range = 14–38).

**Table 1:** Comparison between patients receiving surgical and conservative management.

Variable	All patients	Conservative (*n* = 24)	Surgery (*n* = 101)	*p*-value	VIF
Demographics					
Age (years)	63.54 ± 12.2	60.96 ± 9.29	64.15 ± 12.64	0.25	1.11
Gender, M/F	73/52	12/12	61/40	0.35	1.05
Clinical parameters					
ASIA on admission	79.26 ± 15.25	87.08 ± 7.28	77.4 ± 16.07	0.005	1.79
NRS on admission	6.46 ± 2.27	6.46 ± 2.167	6.47 ± 2.31	0.989	1.07
Radiological features					
Cord T2 hyperintensity	44	8	36	0.831	1.45
OLF	26	3	23	0.265	1.33
>50% contrast enhancement on MRI	49	7	42	0.263	1.15
Radiological quantification					
Length of abscess	54.36 ± 39.44	49.33 ± 21.32	55.56 ± 42.23	0.489	1.23
MSCC	0.289 ± 0.192	0.197 ± 0.171	0.312 ± 0.193	0.006	1.03
Cord area	31.53 ± 1.95	31.75 ± 2.04	31.48 ± 1.93	0.55	1.09

*ASIA*, American Spinal Injury Association; *NRS*, Numerical Rating Scale; *OLF*, ossification of the ligamentum flavum; *MSCC*, maximum spinal cord compression; *VIF*, variance inflation factor.

### Conservative *versus* surgical management

Patients receiving surgical and conservative treatments did not differ by age (60.96 ± 9.29 *vs*. 64.15 ± 12.64, *p* = 0.25) or gender (12/12 *vs*. 51/40, *p* = 0.35). Patients receiving surgery had lower admission ASIA scores (87.08 ± 7.28 *vs*. 77.4 ± 16.07, *p* = 0.005) and greater MSCC (0.312 ± 0.193 *vs*. 0.197 ± 0.171, *p* = 0.006). The remaining analyzed variables, which were not significantly different between the two groups, are listed in **Table 1**.

### Effect of surgical management and time to surgery

Univariate regression (**Table 2**) demonstrated that surgical management had a significant effect on the ASIA recovery rate on the final follow-up. Overall, 58 patients (46.4%) achieved good ASIA recovery, while 67 patients (53.6%) had poor recovery. In the surgical cohort (*n* = 101), the average duration of antibiotic therapy was 17.47 ± 8.1 days, with 42 patients (41.6%) achieving good recovery. Among patients undergoing surgery within 3 weeks (*n* = 73), 35 (47.9%) achieved good recovery compared with only 7 out of 28 patients (25.0%) who received surgery beyond 3 weeks (*p* = 0.032).

**Table 2:** Univariate linear regression analysis of the factors associated with the American Spinal Injury Association (ASIA) motor score at the final follow-up, the ASIA recovery rate, and ΔASIA.

Variable	ASIA final follow-up: *p*-value (*β*)	95%CI	ASIA recovery rate: *p*-value (*β*)	95%CI	ΔASIA: *p*-value (*β*)	95%CI
Demographics						
Age (years)	0.692 (−0.04)	−0.25 to 0.16	0.882 (0)	−0.02 to 0.05	0.474 (0.03)	−0.05 to 0.11
Gender	0.798 (−0.65)	−5.67 to 4.37	0.625 (0.03)	−1.37 to 0.26	0.784 (−0.27)	−2.18 to 1.65
Clinical parameters						
ASIA on admission	<0.001 (0.86)	0.8–0.91	<0.001 (0.001)	−0.03 to 0.02	/	/
NRS on admission	0.588 (−0.3)	−1.39 to 0.79	0.383 (−0.001)	1–1	0.303 (−0.22)	−0.63 to 0.2
Treatment						
Surgery >3 weeks	0.045 (−5.99)	−11.85 to −0.13	0.031 (−0.13)	−0.25 to −0.01	0.365 (1.08)	−1.27 to 3.43
Conservative	0.019 (7.47)	1.27–13.68	0.001 (−0.22)	−0.34 to −0.09	0.84 (−0.25)	−2.74 to −0.09
Radiological features						
Cord T2 hyperintensity	<0.001 (−13.83)	−18.38 to −9.28	<0.001 (−0.24)	−0.47 to 1.21	0.972 (−0.04)	−2.01 to 1.94
OLF	<0.001 (−11.95)	−17.66 to −6.25	<0.001 (−0.2)	−1.1 to 0.9	<0.001 (−4.64)	−6.81 to −2.47
>50% contrast enhancement on MRI	<0.001 (−10.3)	−15.02 to −5.58	<0.001 (−0.28)	−0.82 to 0.84	0.972 (−0.04)	−3.57 to 0.26
Radiological quantification						
Length of abscess	0.474 (0.02)	−0.04 to 0.09	0.239 (0)	0–0.02	0.723 (0)	−0.03 to 0.02
MSCC	<0.001 (−0.25)	−0.42 to −0.03	0.08 (0)	−0.03 to 0.02	0.041 (−0.05)	−0.1 to 0
Cord area	0.936 (−0.05)	−1.33 to 1.22	0.483 (−0.01)	−0.24 to 0.18	0.24 (−0.29)	−0.77 to 0.19

*NRS*, Numerical Rating Scale; *OLF*, ossification of the ligamentum flavum; *MSCC*, maximum spinal cord compression.

### Radiographic parameters

#### Indicators of severity of insult

Univariate regression (**Table 3**) demonstrated that cord T2 hyperintensity, OLF, and >50% contrast on MRI were associated with lower admission ASIA scores (*p* < 0.001, *p* < 0.001, and *p* = 0.002, respectively). Increased MSCC correlated with low ASIA scores (*β* = −0.3, *p* < 0.001). The aforementioned findings suggest that reduced intraspinal-occupying lesions and a more favorable spinal cord condition may be associated with better initial neurological status following SEA.

**Table 3:** Univariate linear regression analysis of the factors associated with the American Spinal Injury Association (ASIA) motor score on admission.

Variable	ASIA on admission: *p*-value (*β*)	95%CI	NRS on admission: *p*-value (*β*)	95%CI
Demographics				
Age (years)	0.541 (−0.07)	−0.29 to 0.15	0.337 (0.02)	−0.02 to 0.05
Gender	0.89 (−0.38)	−5.89 to 5.12	0.179 (−0.56)	−1.37 to 0.26
Radiological features				
Cord T2 hyperintensity	<0.001 (−13.79)	−18.91 to −8.68	0.038 (0.37)	−0.47 to 1.21
OLF	<0.001 (−16.59)		0.842 (−0.1)	−1.1 to 0.9
>50% contrast enhancement on MRI	0.002 (−8.64)	−22.58 to −10.6	0.983 (0.01)	−0.82 to 0.84
Radiological quantification				
Length of abscess	0.438 (0.03)	−0.04 to 0.1	0.099 (0.01)	0–0.02
MSCC	<0.001 (−0.3)	−0.43 to −0.17	0.043 (−0.01)	−0.03 to −0.01
Cord area	0.738 (0.24)	−1.16 to 1.63	0.764 (−0.03)	−0.24 to 0.18

*NRS*, Numerical Rating Scale; *OLF*, ossification of the ligamentum flavum; *MSCC*, maximum spinal cord compression.

#### Predictors of final neurological recovery

As shown in **Tables 2**–**6**, the ASIA scores on the final follow-up were positively correlated with the admission ASIA scores (*β* = 0.86, *p* < 0.001) and were negatively correlated with cord T2 hyperintensity (*β* = −13.83, *p* < 0.001), OLF (*β* = −11.95, *p* < 0.001), >50% enhancement contrast on MRI (*β* = −10.3, *p* < 0.001), and MSCC (*β* = −0.25, *p* < 0.001). Similarly, the ASIA recovery rate was negatively correlated with cord T2 hyperintensity (*β* = −0.24, *p* < 0.001), OLF (*β* = −0.2, *p* < 0.001), and >50% enhancement contrast on MRI (*β* = −0.28, *p* < 0.001).

**Table 4:** Univariate linear regression analysis of the factors associated with the Numerical Rating Scale (NRS) pain score at the final follow-up and the NRS recovery rate.

Variable	NRS at final follow-up: *p*-value (*β*)	95%CI	NRS recovery rate: *p*-value (*β*)	95%CI
Demographics				
Age (years)	0.337 (0.02)	−0.02 to 0.05	0.26 (0)	0.00–0.00
Gender	0.179 (−0.56)	−1.37 to 0.26	0.18 (0.05)	−0.07 to 0.13
Clinical parameters				
ASIA on admission	0.892 (0)	−0.03 to 0.02	0.76 (0)	0.01–0.01
NRS on admission	<0.001 (−0.09)	1–1	<0.001 (−0.09)	−0.03 to 0.01
Treatment				
Surgery >3 weeks	0.925 (0.05)	−0.96 to 1.06	0.864 (0.01)	0.05–0.3
Conservative	0.991 (0.01)	−1.06 to 1.07	0.478 (−0.04)	−0.26 to 0
Radiological features				
Cord T2 hyperintensity	0.036 (0.37)	−0.47 to 1.21	0.387 (−0.04)	−0.32 to −0.16
OLF	0.842 (−0.1)	−1.1 to 0.9	0.771 (−0.01)	−0.28 to −0.04
>50% contrast enhancement on MRI	0.983 (0.01)	−0.82 to 0.84	0.976 (0)	−0.35 to −0.16
Radiological quantification				
Length of abscess	0.099 (0.01)	0–0.02	0.109 (0)	0.00–0.00
MSCC	0.483 (−0.01)	−0.02 to 0.32	0.823 (0)	−0.03 to 0.02
Cord area	0.764 (−0.03)	−0.24 to 0.18	0.865 (0)	−0.03 to 0.02

*ASIA*, American Spinal Injury Association; *OLF*, ossification of the ligamentum flavum; *MSCC*, maximum spinal cord compression.

**Table 5:** Univariate logistic regression analysis of the predictors for good American Spinal Injury Association (ASIA) recovery.

Variable	OR (95%CI)	*p*-value
Demographics		
Age (years)	0.99 (0.96–1.02)	0.532
Gender	1.02 (0.5–2.08)	0.963
Clinical parameters		
ASIA on admission	1.19 (1.12–1.27)	<0.001
NRS on admission	0.98 (0.84–1.15)	0.818
Treatment		
Surgery >3 weeks Conservative	0.36 (0.14–0.96) 2.17 (0.83–5.7)	0.04 0.115
Radiological features		
Cord T2 hyperintensity	0.24 (0.11–0.54)	0.001
OLF	0.1 (0.03–0.37)	<0.001
>50% contrast enhancement on MRI	0.1 (0.04–0.25)	<0.001
Radiological quantification		
Length of abscess	1.01 (1–1.02)	0.209
MSCC	1.02 (1–1.03)	0.107
Cord area	0.97 (0.81–1.16)	0.726

*NRS*, Numerical Rating Scale; *OLF*, ossification of the ligamentum flavum; *MSCC*, maximum spinal cord compression.

**Table 6:** Multivariable logistic regression analysis of predictors for good American Spinal Injury Association (ASIA) recovery (after LASSO variable selection).

Variable	OR (95%CI)	*p*-value
Clinical parameter		
ASIA on admission	1.19 (1.12–1.27)	<0.001
Treatment		
Surgery >3 weeks	0.36 (0.14–0.96)	0.04
Conservative	2.17 (0.83–5.7)	0.115
Radiological features		
Cord T2 hyperintensity	0.24 (0.11–0.54)	0.001
OLF	0.1 (0.03–0.37)	<0.001
>50% contrast enhancement on MRI	0.1 (0.04–0.25)	<0.001

*OLF*, ossification of the ligamentum flavum; *LASSO*, least absolute shrinkage and selection operator.

Analysis of absolute neurological improvement (ΔASIA) demonstrated that the presence of OLF was significantly associated with smaller neurological gains at the final follow-up (*β* = −4.64, 95%CI = −6.81 to −2.47, *p* < 0.001). Increased MSCC was also negatively associated with ΔASIA (*β* = −0.05, 95%CI = −0.10 to 0.00, *p* = 0.041). In contrast, delayed surgery (>3 weeks), cord T2 hyperintensity, and >50% contrast enhancement were not significantly associated with absolute ASIA improvement (all *p* > 0.05). This discrepancy indicates that the association between surgical timing and neurological recovery may be sensitive to the choice of outcome definition. The NRS scores on the final follow-up demonstrated a negative correlation with the admission NRS (*p* < 0.001, *β* = −0.09).

Binary logistic regression for ‘‘good’’ and ‘‘poor’’ ASIA recovery demonstrated that a higher admission ASIA score (OR = 1.19, *p* < 0.001) was associated with good ASIA recovery, cord T2 hyperintensity (OR = 0.24, *p* < 0.001), OLF (OR = 0.1, *p* < 0.001), and >50% enhancement contrast on MRI (OR = 0.1, *p* < 0.001). Prolonged preoperative antibiotic use >3 weeks (OR = 0.36, *p* = 0.04) was associated with poor ASIA recovery. We determined that a threshold value for an admission ASIA score of more than 88 was predictive of a good ASIA recovery rate (AUC = 0.927, 95%CI = 0.876–0.977, sensitivity = 0.81, specificity = 0.925, *p* < 0.001).

To assess the robustness of the timing effect, a sensitivity analysis restricted to surgically treated patients (*n* = 101) was performed. After adjustment for admission ASIA score and relevant MRI variables, delayed surgery (>3 weeks) was associated with a lower likelihood of achieving good neurological recovery (OR = 0.2, 95%CI = 0.07–0.57, *p* = 0.03) (**Table 7**).

**Table 7:** Multivariable logistic regression analysis of the predictors for good neurological recovery in surgically treated patients (sensitivity analysis).

Variable	OR (95%CI)	*p*-value
Clinical parameter		
ASIA on admission	1.2 (1.12–1.29)	<0.001
Treatment		
Surgery >3 weeks	0.2 (0.07–0.57)	0.03
Radiological features		
Cord T2 hyperintensity	0.12 (0.04–0.35)	0.001
OLF	0.15 (0.04–0.37)	0.004
>50% contrast enhancement on MRI	0.11 (0.03–0.29)	<0.001

*ASIA*, American Spinal Injury Association; *OLF*, ossification of the ligamentum flavum.

## Discussion

### Principal findings

The present study aimed to identify robust clinical and radiological predictors of neurological recovery in patients with SEA. By integrating quantitative MRI-derived parameters with clinical management variables, we demonstrated that the admission neurological status, spinal cord signal changes, the degree of canal and cord compression, and the timing of surgical intervention significantly influenced the neurological outcomes. Among these factors, an admission ASIA motor score of >88 emerged as a highly accurate predictor of favorable neurological recovery, while prolonged preoperative antibiotic therapy exceeding 3 weeks was associated with poorer neurological outcomes in the dichotomized analysis. However, this association was not observed with ΔASIA. Given the sensitivity of the timing signal to the outcome definition and the presence of substantial unmeasured confounding, this finding should be regarded as hypothesis-generating rather than definitive.

### MRI-based characteristics of neurological prognosis in SEA

MRI plays a pivotal role in the diagnosis and prognostication of spinal cord pathologies. Signal changes within the spinal cord, particularly the T2-weighted hyperintensity, are widely regarded as surrogate markers of edema, ischemia, and irreversible myelomalacia. In the context of degenerative cervical myelopathy, Arvin et al. (2013) demonstrated that preoperative T2 hyperintensity is correlated with worse baseline neurological function and inferior postoperative recovery, while Kato et al. (2018) further showed that persistent postoperative signal abnormalities portend unfavorable outcomes. Our findings extend these observations to the infectious setting of SEA, confirming that spinal cord T2 hyperintensity is a strong indicator of both severe initial neurological insult and limited recovery potential.

Beyond intramedullary signal changes, we found that OLF and the degree of abscess enhancement on contrast MRI were independently associated with poor neurological outcomes. OLF likely represents a preexisting degenerative narrowing of the spinal canal (Bagga et al., 2023), reducing the tolerance of the spinal cord to acute infectious compression. Similarly, extensive contrast enhancement may reflect a larger inflammatory burden and a more aggressive epidural pathology, contributing to sustained neural compromise. Together, these imaging features function as objective biomarkers of cumulative mechanical and inflammatory injury to the spinal cord.

Nevertheless, caution is warranted when extrapolating evidence from degenerative cervical myelopathy to SEA. Although spinal cord T2 hyperintensity may represent a common imaging marker of neural tissue injury, the underlying mechanisms differ substantially between these conditions. Degenerative cervical myelopathy is characterized by chronic mechanical compression, whereas SEA involves a combination of acute or subacute compression, infection-related inflammation, vascular compromise, and potential neurotoxicity from the infectious process. Therefore, while our findings support the prognostic relevance of T2 hyperintensity in SEA, the biological interpretation of this imaging feature may not be identical to that reported in degenerative spinal disorders. Further studies specifically focused on SEA are needed to validate these associations and clarify their pathophysiological basis.

Quantitative compression metrics were also significantly correlated with the admission ASIA scores and the final neurological outcomes. These parameters have been validated in degenerative and traumatic spinal conditions as reproducible measures of spinal cord compression severity, but have been underutilized in SEA. Our data support their incorporation into routine MRI assessment for risk stratification in this population.

### Timing of surgery and impact of prolonged antibiotic therapy

The optimal timing of surgical decompression in SEA remains controversial, particularly for patients without rapidly progressive neurological deficits (Tang et al., 2002; Davis et al., 2004). While some studies have suggested that certain patients may be safely managed with antibiotics alone, accumulating evidence indicates that delayed surgical intervention may compromise neurological recovery. Luo et al. (2018) reported improved neurological outcomes with earlier operative management in spinal infections, whereas Behmanesh et al. (2020) demonstrated superior functional outcomes and quality of life with early surgery in SEA.

In the present cohort, patients undergoing decompression within 3 weeks of diagnosis achieved significantly higher rates of good neurological recovery compared with those receiving delayed surgery. Importantly, preoperative antibiotic therapy exceeding 3 weeks was associated with poor neurological recovery. This finding may reflect several potential mechanisms, including persistent mechanical compression and ongoing inflammatory injury during prolonged conservative management. However, residual confounding cannot be excluded, and patients undergoing delayed surgery may also have differed in baseline disease characteristics that were not fully captured in our dataset. To further evaluate the robustness of this finding, we performed a sensitivity analysis restricted to surgically treated patients. After adjustment for the admission ASIA motor score and relevant MRI variables, delayed surgery (>3 weeks) remained independently associated with a lower likelihood of achieving good neurological recovery. These findings suggest that the observed association was not solely driven by the differences between surgically and conservatively managed patients.

Notably, surgical treatment demonstrated a negative beta coefficient in multivariate models predicting absolute ASIA motor scores at the final follow-up. This finding should not be interpreted as evidence that surgery itself leads to inferior neurological outcomes. Rather, it reflects confounding by indication (Kyriacou and Lewis, 2016) as the surgically treated patients in this study presented with significantly worse baseline neurological function and more severe radiographic compression compared with patients who were conservatively treated.

As a result, although surgical patients may experience meaningful neurological improvement, their absolute ASIA motor scores at the final follow-up may remain lower than those of patients initially managed conservatively but who had milder deficits at presentation. This interpretation is supported by analyses based on relative recovery metrics, including the ASIA recovery rate and the dichotomized good *versus* poor recovery, in which delayed surgery and prolonged preoperative antibiotic therapy were consistently associated with inferior outcomes. Therefore, the observed negative association between surgery and absolute neurological scores primarily reflects baseline disease severity rather than a detrimental effect of surgical decompression.

### Clinical and radiological predictors for neurological outcomes

The admission neurological status remains one of the most powerful predictors of outcome in SEA. Consistent with prior literature, we found that higher admission ASIA motor scores were strongly associated with superior neurological recovery. The identification of an ASIA motor score threshold >88, with excellent sensitivity and specificity, provides a clinically practical benchmark for early prognostic counseling and treatment planning.

When integrated with radiological indicators such as spinal cord signal change, compression severity, and abscess enhancement, this threshold enables a comprehensive and individualized risk stratification strategy. Patients presenting with preserved neurological function but high-risk imaging features may benefit from earlier surgical intervention, whereas those with favorable imaging and high ASIA scores may be candidates for cautious conservative management under close surveillance.

### Limitations

This study has several limitations. Firstly, as a retrospective single-center cohort study, it is subject to inherent selection bias and confounding by indication. Secondly, although quantitative MRI parameters were independently measured by two experienced radiologists with good inter-observer agreement, some degree of variability cannot be entirely excluded. Thirdly, due to the retrospective nature of the study and the inconsistencies in clinical documentation, several important variables were not comprehensively available, including detailed microbiological data (e.g., culture results and antimicrobial susceptibility), comorbidity burden, specific spinal segmental involvement, appropriateness of empiric antimicrobial therapy, and validated infection severity scores. Positive cultures were obtained in only a subset of patients, and systematic evaluation of antimicrobial appropriateness was not feasible. These factors are well-established prognostic elements in SEA, and their absence may have introduced residual confounding.

Furthermore, surgical timing was determined by clinical decision making rather than randomization, and patients undergoing delayed surgery (>3 weeks) may have differed from those receiving early intervention in terms of clinical stability, comorbidity burden, and response to initial antibiotic therapy. Therefore, the observed association between prolonged preoperative antibiotic therapy and poorer neurological recovery should be interpreted with caution as hypothesis-generating rather than indicative of a definitive causal relationship. Treatment allocation itself also reflected baseline disease severity.

Future prospective, multicenter studies with standardized data collection protocols including routine microbiological profiling, comorbidity indexing, and infection severity assessment are warranted to validate our findings and to better elucidate the interplay between infectious parameters, radiographic features, and neurological recovery.

## Conclusion

This study summarizes the prognostic factors for neurological recovery in SEA. Patients presenting with lower admission ASIA scores, prolonged preoperative antibiotic use, and adverse MRI features (i.e., cord T2 hyperintensity, OLF, and >50% contrast enhancement) are at increased risk of poor recovery. An admission ASIA score of >88 serves as a valuable cutoff for predicting favorable outcomes. Our findings suggest that earlier surgical decompression may be associated with more favorable neurological recovery in appropriately selected patients and provide a framework for preoperative risk stratification to guide individualized management.

## Data Availability

The original contributions presented in the study are included in the article/supplementary material. Further inquiries can be directed to the corresponding authors.
